# Work Function Tuning in Two-Dimensional MoS_2_ Field-Effect-Transistors with Graphene and Titanium Source-Drain Contacts

**DOI:** 10.1038/srep45546

**Published:** 2017-03-30

**Authors:** Seung Su Baik, Seongil Im, Hyoung Joon Choi

**Affiliations:** 1 School of Computational Sciences, Korea Institute for Advanced Study, Seoul 02455, Korea; 2 Department of Physics and IPAP, Yonsei University, Seoul 03722, Korea; 3 Center for Computational Studies of Advanced Electronic Material Properties, Yonsei University, Seoul 03722, Korea

## Abstract

Based on the first principles calculation, we investigate the electronic band structures of graphene-MoS_2_ and Ti-MoS_2_ heterojunctions under gate-voltages. By simultaneous control of external electric fields and carrier charging concentrations, we show that the graphene’s Dirac point position inside the MoS_2_ bandgap is easily modulated with respect to the co-varying Fermi level, while keeping the graphene’s linear band structure around the Dirac point. The easy modulation of graphene bands is not confined to the special cases where the conduction-band-minimum point of MoS_2_ and the Dirac point of graphene are matched up in reciprocal space, but is generalized to their dislocated cases. This flexibility caused by the strong decoupling between graphene and MoS_2_ bands enhances the gate-controlled switching performance in MoS_2_-graphene hybrid stacking-device.

Molybdenum disulfide (MoS_2_)^1^,^2^ and graphene^3^,^4^ are rapidly emerging and already successfully emerged nanoelectronic materials. In many ways, MoS_2_ is compared with its predecessor graphene for their beneficial properties such as outstanding carrier mobility^5^,^6^,^7^,^8^,^9^,^10^, high structural flexibility^3^,^11^, and bandgap modulation under external perturbations^12^,^13^,^14^,^15^,^16^,^17^,^18^,^19^. However, in contrast to gapless graphene, pristine MoS_2_ shows a sizable bandgap of ~1.3 eV in its bulk state, which further increases up to ~1.9 eV upon exfoliating. For this reason, extensive efforts have been carried out to take advantages from each material and combine them into a single device. Prominent accomplishments in such efforts are the synthesis of stacked graphene-MoS_2_ junctions^20^,^21^,^22^,^23^ and their application to field effect transistors (FETs)^24^,^25^,^26^,^27^,^28^,^29^,^30^,^31^,^32^,^33^,^34^,^35^,^36^,^37^,^38^,^39^ in which MoS_2_ and graphene are used as a channel and source(S)-drain(D) electrodes, respectively.

In FETs, an ohmic contact is usually desired for easy current flows between semiconductor and S/D electrodes. To fulfill this requirement, relevant contact searching is primarily focused on avoiding materials with the rectifying responses to forward and backward biases. Reported S/D electrodes on MoS_2_ channel are pure metals such as Ti^5^,^6^,^8^,^40^,^41^,^42^,^43^,^44^, Au^40^,^45^,^46^,^47^,^48^,^49^,^50^, and Sc^42^,^43^, and the most popular material amongst them tends to become Au/Ti deposition followed by a post-annealing process^44^. This choice follows from a prior estimation based on the Schottky-Mott rule^51^,^52^,^53^, which states that the potential barrier height at the interface is given by the energy difference between the semiconductor electron affinity and the metal work function. Because the electron affinity of MoS_2_ is reported as 4.0 eV and the work functions of graphene and Ti are known as 4.5 and 4.3 eV, respectively, MoS_2_-Ti contact is expected to form a smaller Schottky barrier, leading to a more ohmic behavior. Contrary to this expectation, however, graphene-contacted MoS_2_ shows higher on-current and lower off-state behaviors, displaying an on/off ratio over ~7.5 × 10^6^ 29. To understand this seemingly anomalous feature, we first constructed graphene/MoS_2_ and Ti/MoS_2_ stacking structures, and investigated their electronic band structures by using the first-principles density functional method^54^. In this work, we explicitly show the absence of graphene-induced gap-states, which otherwise would cause a Fermi level (E_*F*_) pinning^55^ within the bandgap. We then attribute the sensitive variations of E_*F*_ and work function under gate-voltages to the strong decoupling between graphene and MoS_2_ bands in their heterojunction.

## Computational Methods

To investigate the gate-voltage tuning effects on the graphene(S/D)-MoS_2_ and Ti(S/D)-MoS_2_ FETs, we have performed self-consistent density functional calculations using the SIESTA code^54^. Exchange and correlation were treated with the local density approximation (LDA)^56^. Core electrons were replaced by standard norm-conserving pseudo-potentials^57^ as transformed into fully nonlocal Kleinman-Bylander form^58^. Valence states were described by numerical atomic orbitals of double-ζ plus polarization basis-set to account for the deformation density induced by bond formations. Electronic wavefunctions and charge densities were projected onto a real space grid with an equivalent energy cutoff of 500 Ry. We used 24 × 24 k-grid sampling in the full Brillouin zone (BZ) for the slab systems. To describe naturally n-doped MoS_2_, we performed the virtual crystal approximation (VCA) by replacing 1.3 × 10^−4^ atomic % of sulfur atoms with chlorine atoms. This corresponds to 5.0 × 10^16 ^cm^−3^ electron doping in bulk MoS_2_ as consistent with experimental observations^40^,^59^,^60^,^61^,^62^. After the VCA, additional electron charging effects under gate-voltages were simulated by direct electron addition or subtraction methods. For the graphene stacking on MoS_2_, we considered two different heterostructures with commensurability conditions: (*i*) 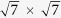 graphene sheet (6.51 Å) was stacked on 2 × 2 MoS_2_ monolayer (6.32 Å) which includes 2.9% lattice contraction of graphene. (*ii*) 4 × 4 graphene sheet (9.84 Å) was adjusted to 3 × 3 MoS_2_ monolayer (9.48 Å), resulting in 3.7% lattice contraction of graphene. For the Ti stacking on MoS_2_, 4-layered Ti lattice (a = 2.95 Å) was placed on 1 × 1 MoS_2_ monolayer (3.16 Å) which includes 6.7% lattice expansion of Ti.

## Results and Discussion

**Figure 1:**
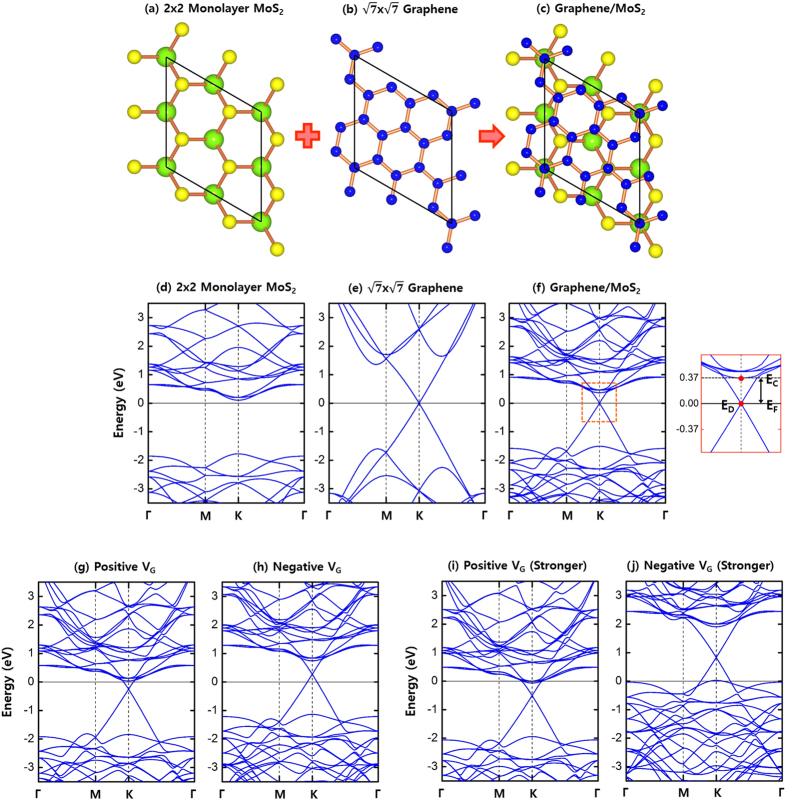
Atomic views and electronic band structures. Top-views of (**a**) 2 × 2 MoS_2_ monolayer, (**b**) 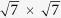 graphene monolayer, and (**c**) 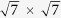-graphene/2 × 2-MoS_2_ heterostructure. Green, yellow, and blue spheres represent Mo, S, and C atoms, respectively. Band structures of (**d**) 2 × 2 MoS_2_ monolayer, (**e**) 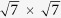 graphene monolayer, and (**f**) 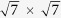-graphene/2 × 2-MoS_2_. Inside the box (orange line), E_*C*_, E_*D*_, and E_*F*_ represent the CBM energy of MoS_2_, the Dirac point energy of graphene, and the Fermi level of the whole system, respectively. Band structures of 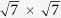 -graphene/2 × 2-MoS_2_ under (**g**) a positive gate-voltage (*E* = + 0.01 V/Å and *n*_*c*_ = 8.7 × 10^12^ cm^−2^), (**h**) a negative gate-voltage (*E* = −0.01 V/Å and *n*_*c*_ = −8.7 × 10^12^ cm^−2^), (**i**) a stronger positive gate-voltage (*E* = + 0.1 V/Å and *n*_*c*_ = 8.7 × 10^13^ cm^−2^), and (**j**) a stronger negative gate-voltage (*E* = −0.1 V/Å and *n*_*c*_ = −8.7 × 10^13^ cm^−2^). The direction of a positive electric field is from MoS_2_ to graphene.

In FETs, gate-voltage (V_G_) always puts into effects of electron charging and electric field variation in a simultaneous way. Once the system reaches a given state of on-current at a specific positive gate-voltage, both direction and strength of current flows are controlled by the drain-voltage (V_D_). Figure 1(f) and (g) show the approaching steps from an ungated case to an on-current stage in 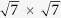-graphene/2 × 2-MoS_2_ heterojuncion. To describe a weak positive (negative) gate-voltage, we have set the external electric field as *E* = ± 0.01 V/Å and the electron charging concentration as *n*_*c*_ = ± 8.7 × 10^12^ cm^−2^. Here the positive and the negative sign represent the positive and the negative V_G_, respectively. To describe stronger gate-voltages, we have increased the electric field and the electron charging concentration by 10 times.

In graphene/MoS_2_ systems, the Schottky barrier height is defined as the energy difference between the conduction band minimum (CBM) energy (E_*C*_) of MoS_2_ in the graphene/MoS_2_ system and the Fermi level (E_*F*_) of the whole system. As seen in Table 1, the Schottky barrier (ΔE = E_*C*_ − E_*F*_) in graphene/MoS_2_ sensitively responds to gate-voltages by decreasing from 0.37 eV (ungated) to 0.03 eV (on-current) in the positive V_G_, and by increasing from 0.37 eV (ungated) to 0.74 eV (off-state) in the negative V_G_. Easily reducible Schottky barrier in positive V_G_ means easily diminishable contact resistance^33^,^34^,^35^,^36^,^37^ between graphene and MoS_2_, which induces large current flows in drain-voltages. In contrast, a large Schottky barrier in negative V_G_ reduces the unprofitable current leakage in off-states and contributes to the high on/off ratio in itself. The working principles of V_D_ and V_G_ are similar to each other, except that V_D_ additionally controls the chemical potential of graphene with respect to that of MoS_2_. Thus, the high sensitivity to gate-voltages involves a similar sensitivity to drain-voltages, resulting in a fast slope increase in the I_D_-V_D_ curve and a corresponding high on/off ratio. Meanwhile, as evaluated in Table 1, the system response to gate-voltages is not completely symmetric due to the shape-change of the Dirac cone caused by the electric field. In the positive V_G_, the Fermi velocity near the Dirac point decreases from 8.38 × 10^5 ^m/s to 8.18 × 10^5 ^m/s, and the Dirac point position becomes closer to the CBM energy of MoS_2_. In contrast, in the negative V_G_, the Fermi velocity increases from 8.38 × 10^5 ^m/s to 8.45 × 10^5 ^m/s, and the Dirac point position becomes far away from the CBM energy of MoS_2_.

The energy difference between the CBM and the Dirac point (E_*C*_ − E_*D*_) is decreased by 0.08 eV from the ungated case (0.37 eV) to the positive V_G_ (0.29 eV), and increased by 0.10 eV from the ungated case (0.37 eV) to the negative V_G_ (0.47 eV). This slightly asymmetric response can be assured by the energy difference between the Fermi level and the Dirac point (E_*F*_ − E_*D*_); increment by 0.25 eV from the ungated case to the positive V_G_, and decrement by 0.27 eV from the ungated case to the negative V_G_. As the gate-voltages increase more, the asymmetry increases further. Shown in Fig. 1(i) and (j) are the band offsets under the strong positive and negative gate-voltages (*E* = ± 0.1 V/Å and *n*_*c*_ = ±8.7 × 10^13^ cm^−2^). Intriguingly, Fig. 1(j) shows a theoretical possibility that the conduction type of real systems may transform from electrons to holes under a strong enough negative V_G_.

**Figure 2:**
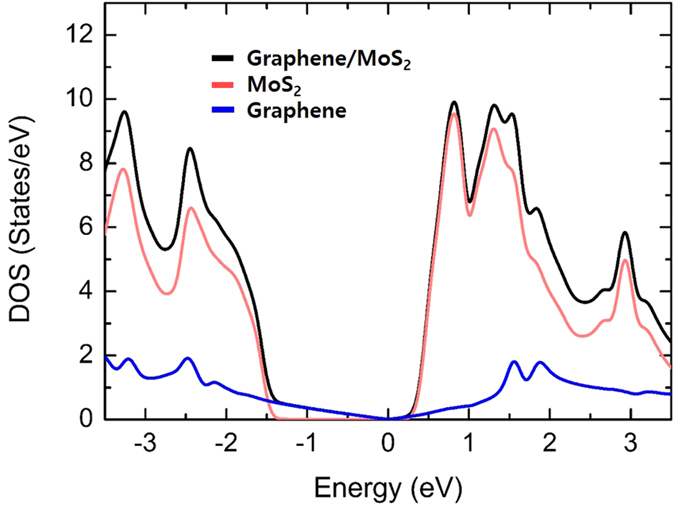
Total and partial density of states (DOS) of 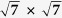-graphene/2 × 2-MoS2 system. Black line is the total DOS of the graphene/MoS_2_ system. Pink line is the partial DOS of MoS_2_ in the graphene/MoS_2_ system. Blue line is the partial DOS of graphene in the graphene/MoS_2_ system.

The band structures shown in Fig. 1(d–j) provide two key features on the flexibility of graphene bands: (*i*) We clearly see the absence of graphene-induced gap-states inside the MoS_2_ bandgap, which would have a flat-band form in k-space and absorb electrons from metals on stacking. This failure in forming the gap-states unlocks the Fermi level pinning and preferably makes the graphene bands flexible. As shown in Fig. 2, the absence of graphene-induced gap-states is confirmed by the clean density of states (DOS) without extra peaks near the Fermi level. (*ii*) The linear shape of graphene bands around the Dirac point remains intact and the linearity extends over one electron-volt from the Dirac point with a negligible band mixing between graphene and MoS_2_. This weak interaction is evidenced from the relatively small binding energy of −0.59 eV and the large equilibrium distance of ~3.23 Å between MoS_2_ and graphene^24^,^25^. In another way, positive electric fields play a role of shifting up the Dirac point toward a higher energy, so if we increase the electric field over ~0.5 V/Å without the electron charging, the Dirac point moves up above the CBM, making the conical vicinity of the Dirac point empty, which then forces E_*F*_ to be located above the CBM to compensate this charge depletion. Even in this extreme electric field, the linearity is retained around the Dirac point, indicating the strong decoupling between graphene and MoS_2_ bands.

**Figure 3:**
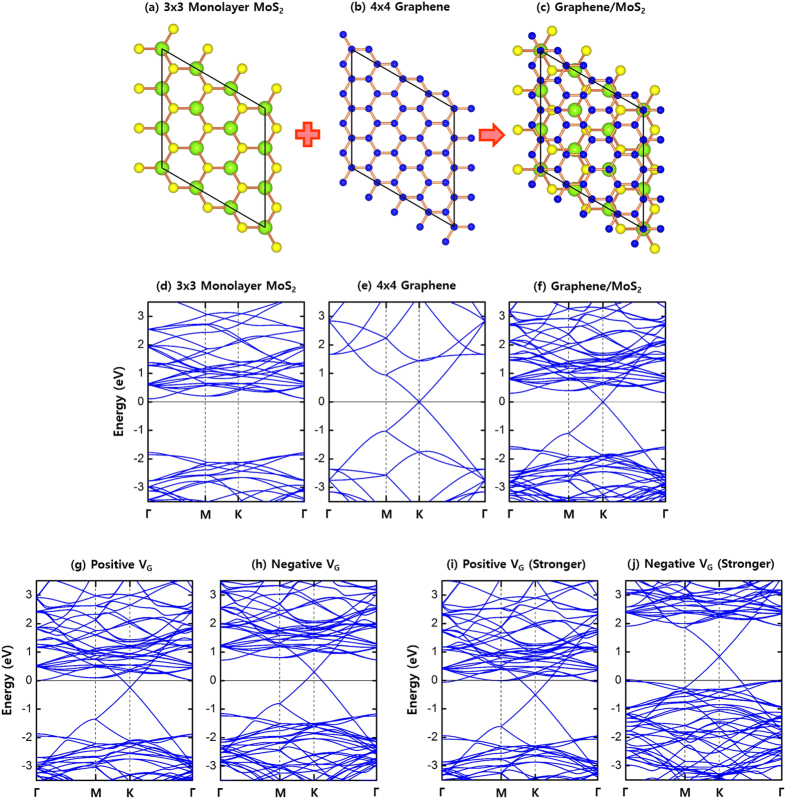
Atomic views and electronic band structures. Top-views of (**a**) 3 × 3 MoS_2_ monolayer, (**b**) 4 × 4 graphene monolayer, and (**c**) 4 × 4-graphene/3 × 3-MoS_2_ heterostructure. Green, yellow, and blue spheres represent Mo, S, and C atoms, respectively. Band structures of (**d**) 3 × 3 MoS_2_ monolayer, (**e**) 4 × 4 graphene monolayer, and (**f**) 4 × 4-graphene/3 × 3-MoS_2_ heterostructure. Band structures of 4 × 4-graphene/3 × 3-MoS_2_ heterostructure under (**g**) a positive gate-voltage (*E* = + 0.01 V/Å and *n*_*c*_ = 8.7 × 10^12^ cm^−2^), (**h**) a negative gate-voltage (*E* = −0.01 V/Å and *n*_*c*_ = −8.7 × 10^12^ cm^−2^), (**i**) a stronger positive gate-voltage (*E* = +0.1 V/Å and *n*_*c*_ = 8.7 × 10^13^ cm^−2^), and (**j**) a stronger negative gate-voltage (*E* = −0.1 V/Å and *n*_*c*_ = −8.7 × 10^13^ cm^−2^). The direction of a positive electric field is from MoS_2_ to graphene.

Thus far, all analyses were performed for the case with the CBM of MoS_2_ and the Dirac point of graphene coincided at the special k-point K. To extend the validity to more general cases where the CBM point and the Dirac point are mismatched, we consider the supercells of 4 × 4 graphene stacked on 3 × 3 MoS_2_. In this new stacking, the CBM point is moved to Γ whereas the Dirac point is still located at K as shown in Fig. 3. We see that all features previously analyzed in Fig. 1 are similarly exhibited in Fig. 3. The band lineups in 4 × 4-graphene/3 × 3-MoS_2_ reproduce sensitive and asymmetric responses to gate-voltages. Also, we see that graphene-induced gap-states do not appear, and the linear dispersion around the Dirac point remains intact on stacking and in onward applications of gate-voltages, leading to the strong decoupling between graphene and MoS_2_ bands. Thus, Fig. 3 confirms that the flexibility of graphene bands is not confined to the special cases where the CBM and Dirac points are matched up in reciprocal space, but is generalized to their dislocated cases.

**Figure 4:**
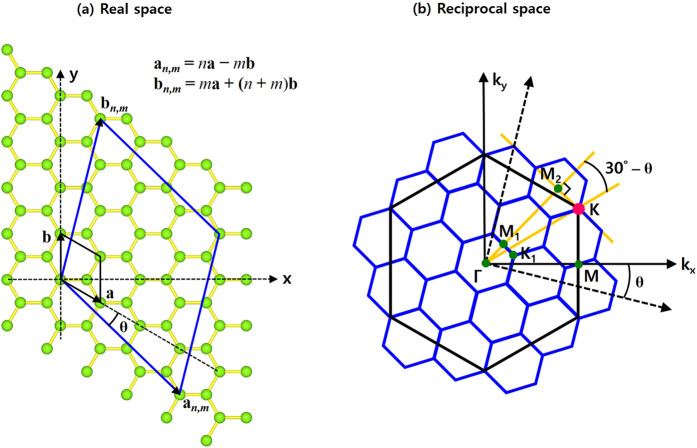
Mapping of special k-points. (**a**) Lattice vectors of a general hexagonal supercell in real space. ***a***_*m,n*_ = *m**a*** − *n**b*** and ***b***_*m,n*_ = *n**a*** + (*m* + *n)**b***, where ***a*** and ***b*** are the lattice vectors of 1 × 1 hexagonal unit cell: 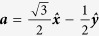 and 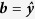. (**b**) Location of special k-points in reciprocal space. The geometric condition 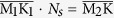 gives rise to the integer value of 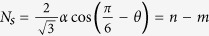, where 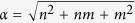. If *N*_*s*_ is a multiple of three, the K point of the unit cell Brillouin zone (BZ) coincides with the Γ point of the supercell BZ. Otherwise, it coincides with the K point of the supercell BZ.

Before going to Ti stacking on MoS_2_, we here analyze the position movement of special k-points from the 1 × 1 hexagonal unit cell to a general hexagonal supercell. Figure 4(a) shows the general hexagonal lattice in real space. The supercell lattice vectors (blue lines) are expressed as ***a***_*m,n*_ = *m**a*** − *n**b*** and ***b***_*m,n*_ = *n**a*** + (*m* + *n)**b***, where 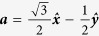 and 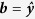 are the lattice vectors of 1 × 1 hexagonal unit cell (black lines). In Fig. 4(b), we can see how the special k-points of general supercells (blues lines) are mapped onto those of 1 × 1 unit cell (black lines) in reciprocal space. In k-space, the length of supercell lattice vector is contracted by the factor,


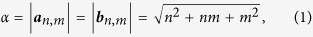


and the geometric condition 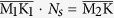 results in the integer value of *N*_*s*_,


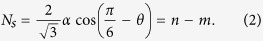


The location of special k-point K for a general hexagonal supercell is determined by the conditions,


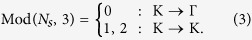


**Figure 5:**
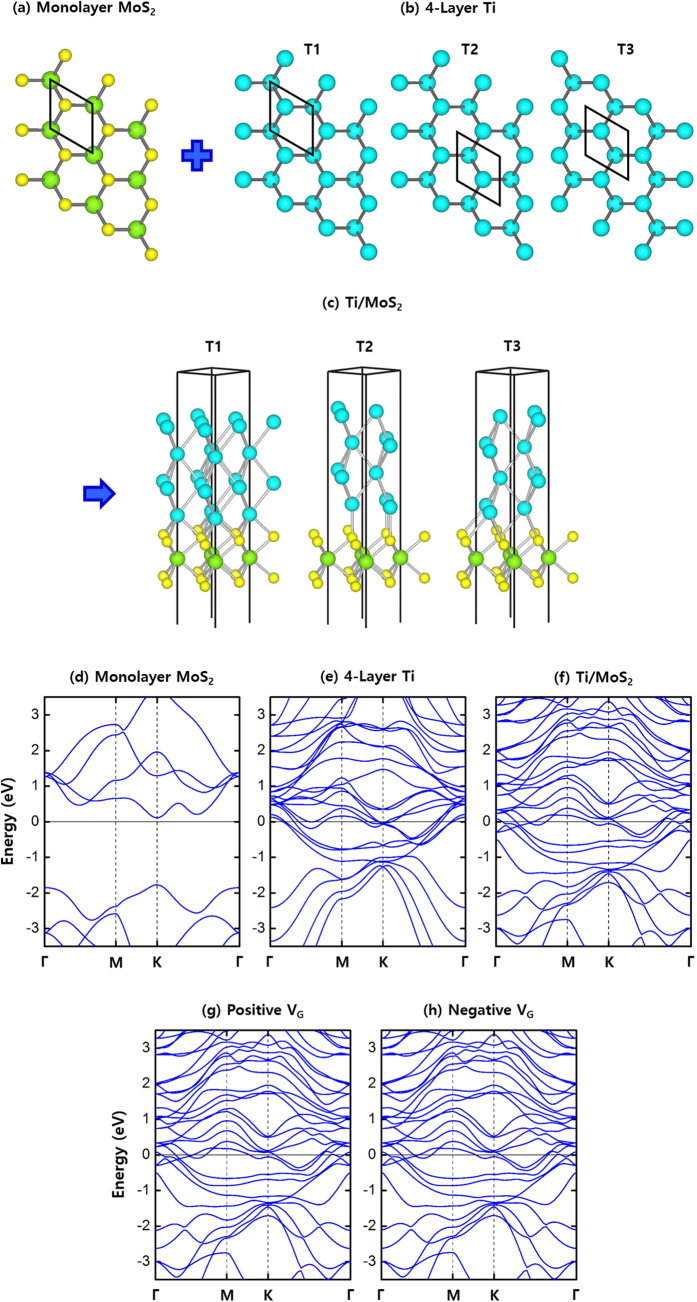
Atomic views and electronic band structures. (**a**) Top-view of monolayer MoS_2_. (**b**) Top-views of 4-layer Ti slabs with three different origin choices, denoted as T1, T2, and T3. (**c**) Ti/MoS_2_ heterostructures. (**d**) Band structure of 1 × 1 MoS_2_ monolayer. (**e**) Band structure of 4-layer Ti. Band structures of Ti/MoS_2_ in the T1 configuration (**f**) without the gate-voltage, (**g**) under a positive gate-voltage (*E* = + 0.01 V/Å and *n*_*c*_ = 8.7 × 10^12^ cm^−2^), and (**h**) under a negative gate-voltage (*E* = −0.01 V/Å and *n*_*c*_ = –8.7 × 10^12^ cm^−2^). The direction of a positive electric field is from MoS_2_ to Ti.

In striking contrast to graphene/MoS_2_, the band offsets in Ti/MoS_2_ show very insensitive behaviors to gate-voltages. To see the differences between graphene/MoS_2_ and Ti/MoS_2_, we constructed three different Ti/MoS_2_ stacking configurations (T1, T2, and T3) as shown in Fig. 5(a–c). Among them, T1 configuration has the lowest total energy, but all the band structures of T1, T2, and T3 configurations are very similar to one another. Figure 5(f–h) show the band structures of T1 configuration. Distinct from graphene/MoS_2_, Fig. 5(f) shows that Ti/MoS_2_ has a strong band mixing along the Γ-M-K-Γ line in the energy range from −1.0 to 1.0 eV. This strong interaction is expected from a short equilibrium distance ~1.60 Å between Ti and MoS_2_, which is less than half the distance ~3.23 Å between graphene and MoS_2_. The binding energy between 2 × 2-Ti and 2 × 2-MoS_2_ is found to be −8.32 eV, which indicates that Ti and MoS_2_ stick together 14 times stronger than graphene and MoS_2_. Comparing Fig. 5(f) with Fig. 5(g) and (h), we see that the position and the shape of Ti bands exhibit negligible changes under V_G_ (*E* = ±0.01 V/Å and *n*_*c*_ = ±8.7 × 10^12^ cm^−2^). Even when we increase the gate-voltages by 10 times (*E* = ±0.1 V/Å and *n*_*ch*_* *=* *±8.7 × 10^13^ cm^−2^), the relative positions of Ti bands are not significantly altered from those in Fig. 5(g) and (h), implying that the intrinsic small Schottky barrier at the interface remains nearly constant without diminishing or rising under the strong gate-voltage. This insensitivity to the gate-voltage is also found in other ohmic metals such as Au (See Supplementary Information).

In conclusion, by simultaneous control of external electric fields and electron charging concentrations, we have compared the band offsets in graphene (S/D)-MoS_2_ with those in Ti(S/D)-MoS_2_. Under gate-voltages, the Dirac point positioning inside the MoS_2_ bandgap is easily modulated with respect to the co-varying Fermi level, while the linearity of graphene bands remains intact around the Dirac point. Band lineups in graphene/MoS_2_ explicitly confirms the absence of graphene-induced gap-states, which is thought to prevent the Fermi level pinning and preferably make graphene bands flexible. In contrast to Ti-MoS_2_, graphene-MoS_2_ interactions are very weak as evidenced from the small binding energy of −0.59 eV and the large equilibrium distance between graphene and MoS_2_ ~3.23 Å, which is more than twice the equilibrium distance ~1.60 Å between Ti and MoS_2_. In effect, the strong decoupling between graphene and MoS_2_ bands causes a high sensitivity to gate-voltages. We respectively analyzed the fast increasing on-current and the steadily maintained (or lowered) off-current states, which originate from such a sensitive work function tuning of graphene under positive and negative V_G_; such work function tuning, in sum, leads to the high on/off ratio in graphene/MoS_2_.

## Additional Information

**How to cite this article**: Baik, S. S. *et al*. Work Function Tuning in Two-Dimensional MoS_2_ Field-Effect-Transistors with Graphene and Titanium Source-Drain Contacts. *Sci. Rep.* **7**, 45546; doi: 10.1038/srep45546 (2017).

**Publisher's note:** Springer Nature remains neutral with regard to jurisdictional claims in published maps and institutional affiliations.

## Supplementary Material

Supplementary Information

## Figures and Tables

**Table 1:** Work function variations in positive and negative gate-voltages (V_G_) in graphene/MoS_2_.

ΔE (eV)	Ungated	Positive V_G_	Negative V_G_
E_*C*_ − E_*D*_	0.37	0.29	0.47
E_*C*_ − E_*F*_	0.37	0.03	0.74
E_*F*_ − E_*D*_	0.00	0.25	−0.27

V_G_ variations are simulated with the external electric field of *E* = ±0.01 V/Å and the electron charging concentration of ±8.7 × 10^12^ cm^−2^, which corresponds to ±0.03 electron charging in the 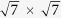-graphene/2 × 2-MoS_2_ supercell. E_*C*_, E_*D*_, and E_*F*_ represent the CBM energy of MoS_2_, the Dirac point energy of graphene, and the Fermi level of the whole system, respectively.
