# Supplementary material for: Work Function Tuning in Two-Dimensional MoS2 Field-Effect-Transistors with Graphene and Titanium Source-Drain Contacts
Source: Sci Rep. 2017 Mar 30;7:45546. doi: 10.1038/srep45546 (PMC5371988; doi:10.1038/srep45546)
Supplement: Supplementary Information [file srep45546-s1.pdf]

Supplementary Information for

Work Function Tuning in Two-Dimensional MoS_2 Field-Effect-Transistors with Graphene and Titanium Source-Drain Contacts

Seung Su Baik[1*], Seongil Im[2], and Hyoung Joon Choi[2,3†]

[1] School of Computational Sciences, Korea Institute for Advanced Study, Seoul 02455, Korea

[2] Department of Physics and IPAP, Yonsei University, Seoul 03722, Korea

[3] Center for Computational Studies of Advanced Electronic Material Properties, Yonsei University, Seoul 03722, Korea

*Email: ssbaik@kias.re.kr

†Email: h.j.choi@yonsei.ac.kr

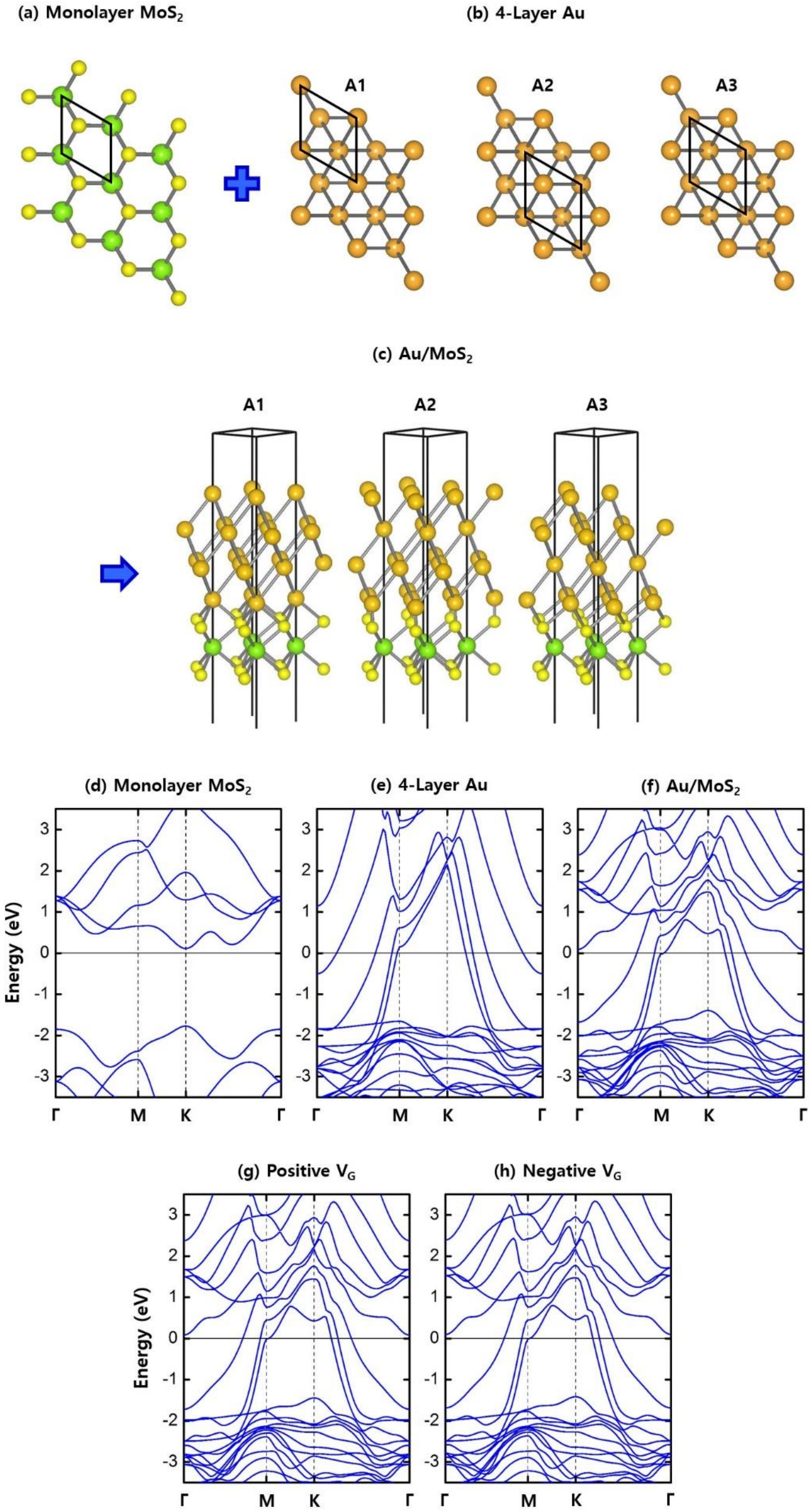
(a) Monolayer MoS2
(b) 4-Layer Au
A1
A2
A3
(c) Au/MoS2
A1
A2
A3
(d) Monolayer MoS2
(e) 4-Layer Au
(f) Au/MoS2
Energy (eV)
3
2
1
0
-1
-2
-3
Γ
M
K
Γ
(g) Positive VG
(h) Negative VG

Figure S1. Atomic views and electronic band structures. (**a**) Top-view of monolayer MoS_2. (**b**) Top-views of 4-layer Au slabs with three different origin choices, denoted as A1, A2, and A3. (**c**) Au/MoS_2 heterostructures. (**d**) Band structure of 1×1 MoS_2 Monolayer. (**e**) Band structure of 4-layer Au. Band structures of Au/MoS_2 in the A2 configuration (**f**) without the gate-voltage, (**g**) under a positive gate-voltage (E = +0.01V/Å and $n_c = 8.7\times10^{12}$ cm^{-2}), and (**h**) under a negative gate-voltage (E = −0.01V/Å and $n_c = -8.7\times10^{12}$ cm^{-2}). The direction of a positive electric field is from MoS_2 to Au.

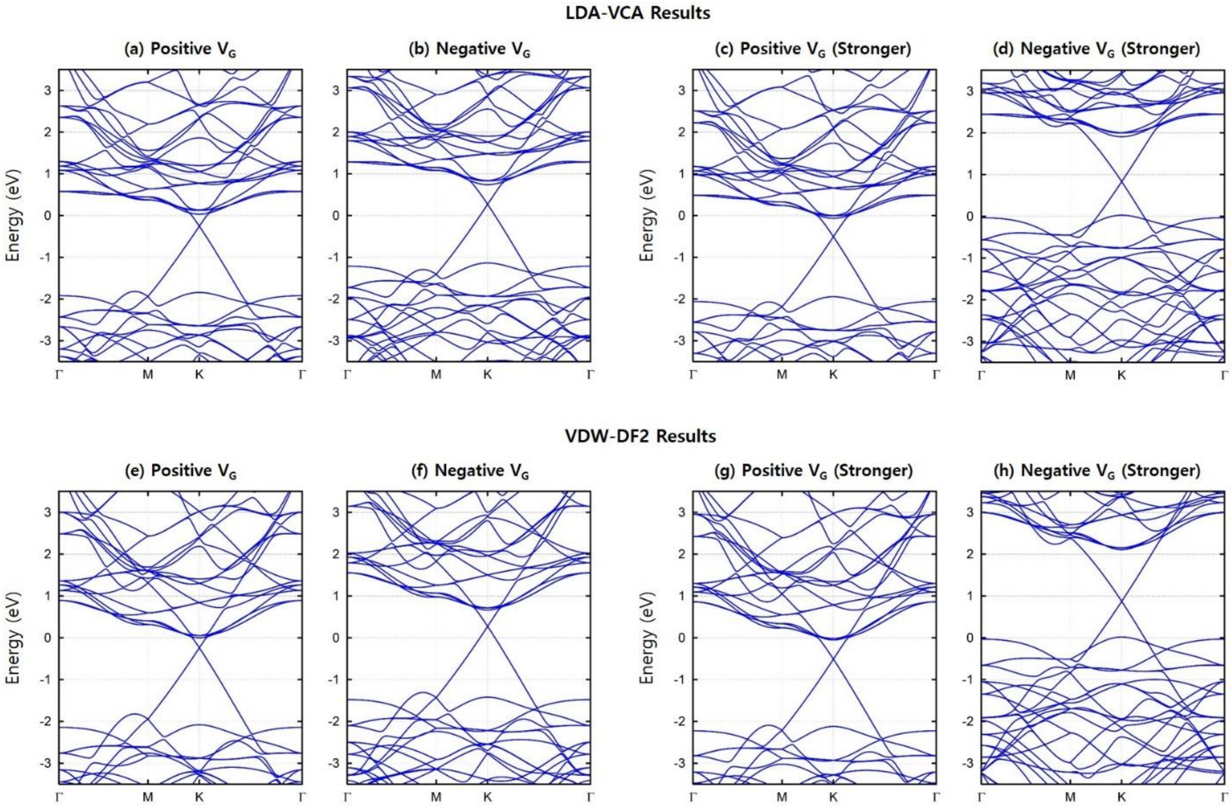

Figure S2. Band structures of $\sqrt{7} \times \sqrt{7}$-graphene/2×2-MoS_2 system. LDA-VCA results under (**a**) a positive V_G (E = +0.01 V/Å and n_c = 8.7×10^{12} cm^{-2}), (**b**) a negative V_G (E = –0.01 V/Å and n_c = –8.7×10^{12} cm^{-2}), (**c**) a stronger positive V_G (E = +0.1 V/Å and n_c = 8.7×10^{13} cm^{-2}), and (**d**) a stronger negative V_G (E = –0.1 V/Å and n_c = –8.7×10^{13} cm^{-2}). Van der Waals (VDW)-DF2 [Ref. S1] results under (**e**) a positive V_G (E = +0.01 V/Å and n_c = 8.7×10^{12} cm^{-2}), (**f**) a negative V_G (E = –0.01 V/Å and n_c = –8.7×10^{12} cm^{-2}), (**g**) a stronger positive V_G (E = +0.1 V/Å and n_c = 8.7×10^{13} cm^{-2}), and (h) a stronger negative V_G (E = –0.1 V/Å and n_c = –8.7×10^{13} cm^{-2}). The direction of a positive electric field is from MoS_2 to graphene. In the VDW-DF2 calculation, the equilibrium distance between graphene and MoS_2 is found to be 3.52 Å.

[S1] K. Lee, E. Murray, L. Kong, B. I. Lundqvist and D. C. Langreth, Phys. Rev. B 82, 081101 (2010).

Examples of special k-point mapping

n	m	α	θ	N_s	MOD(N_s, 3)	K moves to
1	1	1.7321	30.0000	0	0	Γ
2	1	2.6458	19.1066	1	1	K
3	1	3.6056	13.8979	2	2	K
4	1	4.5826	10.8934	3	0	Γ
5	1	5.5678	8.9483	4	1	K
6	1	6.5574	7.5891	5	2	K
7	1	7.5498	6.5868	6	0	Γ
8	1	8.5440	5.8175	7	1	K
9	1	9.5394	5.2087	8	2	K
10	1	10.5357	4.7150	9	0	Γ
11	1	11.5326	4.3066	10	1	K
12	1	12.5300	3.9632	11	2	K
13	1	13.5277	3.6705	12	0	Γ
14	1	14.5258	3.4180	13	1	K
15	1	15.5242	3.1979	14	2	K
16	1	16.5227	3.0045	15	0	Γ
17	1	17.5214	2.8331	16	1	K
18	1	18.5203	2.6802	17	2	K
19	1	19.5192	2.5429	18	0	Γ
20	1	20.5183	2.4190	19	1	K
21	1	21.5174	2.3066	20	2	K
22	1	22.5167	2.2042	21	0	Γ
23	1	23.5160	2.1105	22	1	K
24	1	24.5153	2.0244	23	2	K

n	m	α	θ	N_s	**MOD(N_s, 3)**	**K moves to**
2	2	3.4641	30.0000	0	0	Γ
3	2	4.3589	23.4132	1	1	K
4	2	5.2915	19.1066	2	2	K
5	2	6.2450	16.1021	3	0	Γ
6	2	7.2111	13.8979	4	1	K
7	2	8.1854	12.2163	5	2	K
8	2	9.1652	10.8934	6	0	Γ
9	2	10.1489	9.8264	7	1	K
10	2	11.1355	8.9483	8	2	K
11	2	12.1244	8.2132	9	0	Γ
12	2	13.1149	7.5891	10	1	K
13	2	14.1067	7.0527	11	2	K
14	2	15.0997	6.5868	12	0	Γ
15	2	16.0935	6.1784	13	1	K
16	2	17.0880	5.8175	14	2	K
17	2	18.0831	5.4964	15	0	Γ
18	2	19.0788	5.2087	16	1	K
19	2	20.0749	4.9496	17	2	K
20	2	21.0713	4.7150	18	0	Γ
21	2	22.0681	4.5016	19	1	K
22	2	23.0651	4.3066	20	2	K
23	2	24.0624	4.1278	21	0	Γ
24	2	25.0599	3.9632	22	1	K
25	2	26.0576	3.8113	23	2	K

n	m=n	α	θ	N_s	MOD(N_s, 3)	K moves to
1	1	1.7321	30	0	0	Γ
2	2	3.4641	30	0	0	Γ
3	3	5.1962	30	0	0	Γ
4	4	6.9282	30	0	0	Γ
5	5	8.6603	30	0	0	Γ
6	6	10.3923	30	0	0	Γ
7	7	12.1244	30	0	0	Γ
8	8	13.8564	30	0	0	Γ
9	9	15.5885	30	0	0	Γ
10	10	17.3205	30	0	0	Γ
11	11	19.0526	30	0	0	Γ
12	12	20.7846	30	0	0	Γ
13	13	22.5167	30	0	0	Γ
14	14	24.2487	30	0	0	Γ
15	15	25.9808	30	0	0	Γ
16	16	27.7128	30	0	0	Γ
17	17	29.4449	30	0	0	Γ
18	18	31.1769	30	0	0	Γ
19	19	32.9090	30	0	0	Γ
20	20	34.6410	30	0	0	Γ
21	21	36.3731	30	0	0	Γ
22	22	38.1051	30	0	0	Γ
23	23	39.8372	30	0	0	Γ
24	24	41.5692	30	0	0	Γ
